# *Dirofilaria immitis*: Genotyping Randomly Selected European Clinical Samples and USA Laboratory Isolates with Molecular Markers Associated with Macrocyclic Lactone Susceptibility and Resistance

**DOI:** 10.3390/pathogens11080934

**Published:** 2022-08-18

**Authors:** Emily Curry, Donato Traversa, Elena Carretón, Laura Kramer, Heinz Sager, Lisa Young, Roger Prichard

**Affiliations:** 1 Institute of Parasitology, McGill University, Montreal, QC H9X 3V9, Canada; 2 Faculty of Veterinary Medicine, University of Teramo, 64100 Teramo, Italy; 3 Faculty of Veterinary Medicine, University of Las Palmas de Gran Canaria, 35001 Las Palmas, Spain; 4 Department of Veterinary Science, University of Parma, 43126 Parma, Italy; 5 Elanco Tiergesundheit AG, CH-4058 Basel, Switzerland; 6 Elanco Animal Health, Greenfield, IN 46140, USA

**Keywords:** *Dirofilaria immitis*, dirofilariosis, macrocyclic lactones, susceptibility, resistance, molecular markers, single nucleotide polymorphism

## Abstract

*Dirofilaria immitis* is a parasitic nematode and causes dirofilariosis, a potentially fatal pulmonary infection which primarily infects canids. Dirofilariosis infections are controlled via prophylactic macrocyclic lactone (ML) regimens. Recent evidence has confirmed the development of ML-resistant isolates in the USA, which are genetically distinct from wildtype populations. Single nucleotide polymorphisms (SNP) associated with ML-resistant phenotypes were clinically validated in USA populations. In this study, 3 USA laboratory-maintained isolates (Berkeley, Georgia II, and WildCat) and 11 randomly selected European clinical samples from 7 hosts were analyzed. The samples tested were fresh microfilaria (mf) in blood or adult worms preserved in ethanol. The samples underwent MiSeq sequencing of the top 9 SNP associated with ML resistance. The results provide the first genotypic analysis of the three USA laboratory-maintained isolates and any European samples. The European clinical samples show no genomic evidence of ML resistance based on the 9 SNP. The early adoption of genotyping of clinical *D. immitis* samples could provide an early indication of the potential development of ML resistance and aid to distinguish clinical cases of heartworm infection due to ML resistance from those due to a lack compliance with the recommended treatments, as has been seen in North America.

## 1. Introduction

*Dirofilaria immitis* is a veterinary parasitic filarial nematode and the cause of dirofilariosis, a potentially fatal pulmonary infection which primarily affects canids, with humans occasionally acting as an incidental host. Macrocyclic lactones (MLs) were first approved as a monthly prophylactic treatment for dirofilariosis in 1987 and remain the standard of care [1,2,3,4,5]. The MLs are used at extremely low dose rates; in *D. immitis*, the MLs target and kill the infective L3 and developing L4 larvae. At slightly higher concentrations, the MLs can reduce the fecundity of adults for up to 6 months and help clear blood-circulating microfilariae (mf). This class of drugs has been used as an effective and generally safe prophylactic treatment for preventing dirofilariosis. Complaints of heartworm preventive product ineffectiveness were brought to the USA FDA Center for Veterinary Medicine (FDA/CVM) as early as 1998, only 11 years after being placed on the market. In 2005, ML drug loss of efficacy (LOE) cases, documented in known dirofilariosis hotspots throughout the southern USA, were brought to public attention [6]. The heritability of ML resistant isolates was established in 2014 by experimentally infecting laboratory dogs with *D. immitis* field LOE isolates [7].

Whole genome analysis elucidated single nucleotide polymorphisms (SNPs) associated with a resistant phenotype [8,9,10]. The 10 SNPs which best differentiated the ML-resistant phenotype from the ML-susceptible phenotype were selected for analysis in clinical dirofilariosis infections collected from the continental USA [11]. A significant correlation of the SNP loci frequencies and the moxidectin microfilaricidal response phenotype was observed in 9 of the 10 SNPs [11]. The clinical validation of the molecular markers for ML resistance in *D. immitis* provides the first genetic test to confirm the development of ML-resistant isolates which are genetically distinct from wildtype populations. These markers can be used to differentiate between ML-resistant *D. immitis* isolates versus cases of opportunistic infections caused by lack of compliance, inadequate administration, or no history of ML use.

The prevalence of *Dirofilaria* infections is on the rise in Europe [12]. The rise in dirofilariosis infections is likely the result of increased temperatures due to climate change and increased movement of companion animals across borders [13,14,15]. Hundreds of thousands of dogs are relocated internationally each year in Europe, with more than 300,000 entering the United Kingdom via the EU Pet Travel Scheme (PETS) alone [16]. Large numbers of dogs are also relocated throughout North America, with a recent report from Canada demonstrating that dogs originating in USA are positive for heartworm at double the frequency of Canadian dogs [17]. *Dirofilaria immitis* infections were reported in 109 dogs in Austria, with the dogs originating from Hungary, Greece, the western Balkans, the Iberian Peninsula, Romania, USA, or Bulgaria [18]. As temperatures continue to rise *D. immitis* infections are expected to spread north from the Mediterranean [19]. Autochthonous transmission of canine dirofilariosis has encroached on regions previously untouched by naturally occurring infections such as Hungary and Balkan countries [20,21,22,23]. Given the geographical expansion of dirofilariosis it will be important to monitor the effectiveness of MLs in Europe and the possible emergence of resistance. To date, there is minimal published information on the genetic makeup and diversity of European isolates, with Laidoudi et al. [24] finding a single common haplotype present in European samples analyzed. In the current study, we analyze the prevalence of the validated North American SNP markers associated with susceptibility and resistance to MLs in European clinical samples.

## 2. Results

**Figure 1:**
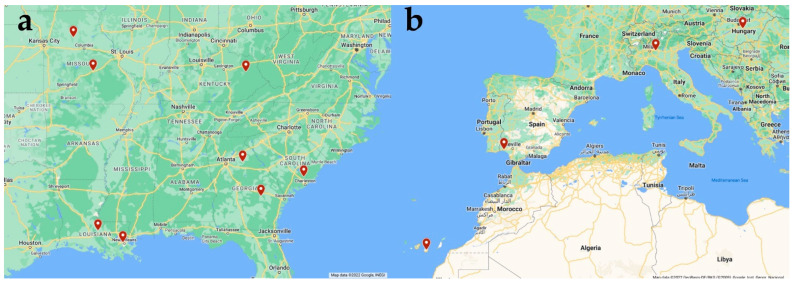
(**a**) Distribution of USA laboratory-maintained isolates received from TRS Laboratories (**b**) Distribution of European clinical samples re-ceived from Italy, Hungary, Spain, and the Canary Islands. The maps were created using Google Maps, accessed 17 August 2022.

The alternate allele frequencies were calculated from the read frequencies using BVAtools in comparison to the *D. immitis* reference genome nDi.2.2 (Dataset S1). The SNP markers were ordered based their individual performance identified with MetaboAnalyst using the Random Forest algorithm, as defined by Ballesteros et al. [11]. The average alternative allele frequency for each of the 9 SNP positions were plotted in a heatmap for the 3 USA laboratory-maintained isolates and the 11 European samples (Figure 1).

**Figure 2:**
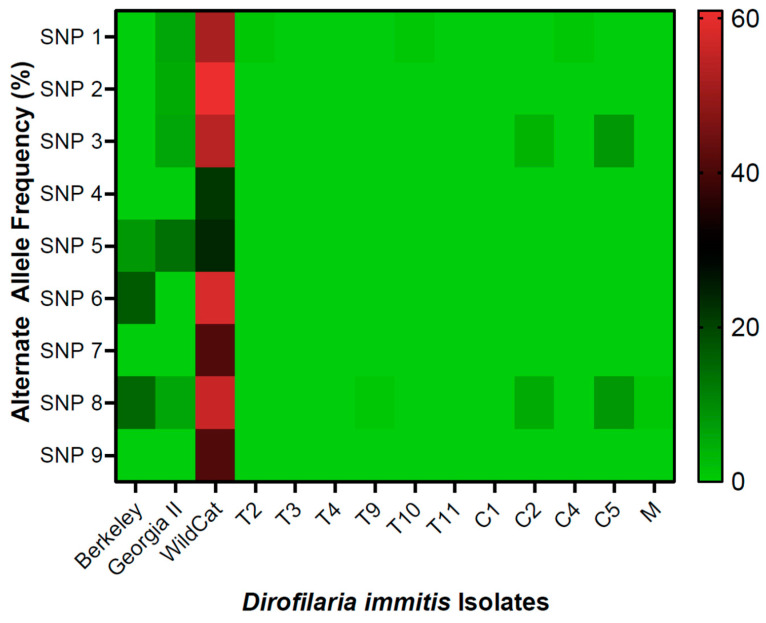
Heat map of the alternate allele frequency of the 9 SNP molecular markers, for the 3 USA laboratory-maintained isolates, Berkeley, Georgia II, and WildCat; and the 11 European clinical samples, T2, T3, T4, T9, T10, T11, C1, C2, C4, C5, and M. The alternative allele frequencies for the 9 SNP molecular markers were prepared in comparison to the *D. immitis* reference genome nDi.2.2.

The WildCat isolate carried higher frequencies of the alternate nucleotide for all SNP markers in the range 22–61%, with a genotype comparable to previously characterized ML-resistant USA laboratory-maintained isolates, such as JYD-34 and Metairie (Figure 2) [9].

The Berkeley isolate displayed some allele alteration in comparison to the reference genome at SNPs 5, 6, and 8 ranging from 8–17% at these three sites (Figure 2). The Georgia II isolate displayed low levels of alternate alleles from 5 to 14% at 5 SNP sites, specifically, 2, 4, 5, 6, and 8 (Figure 2). The 11 European samples displayed genotypes consistent with ML susceptibility. Two European samples C2 and C5 each showed a low-level presence of an alternate allele at two SNP sites. Sample C2 had an alternate allele frequency of 4% at SNP 3 and 5% at SNP 8 (Figure 2). Sample C5 had an alternate allele frequency of 8% at SNP 3 and 8% at SNP 8. Despite these slight alterations in samples C2 and C5 when compared to the reference genome nDi.2.2, the European samples were closely aligned to the reference genome [9].

## 3. Discussion

Previous research indicates an increase in *D. immitis* infections in the USA [25,26]. ML-resistant isolates, genetically distinct from the wildtype population, have been confirmed and documented in the southern USA during the last decade. This is not unexpected after long-term and widespread use of prophylactic MLs [7,8,9,10,11]. The current study completed the first genomic analysis of the USA laboratory-maintained Berkeley and Georgia II, and WildCat isolates, as well as 11 European clinical samples collected from 7 canine hosts which were randomly selected from dirofilariosis endemic regions.

The Berkeley, Georgia II, and WildCat isolates were three isolates used to validate the efficiency of the milbemycin oxime based Credelio Plus™ in in vivo chemoprophylactic studies. The Berkeley and Georgia II isolates demonstrated a ML-susceptible phenotype with 100% parasite clearance as defined by the FDA/CVM and the European Medicines Agency (EMA). The WildCat isolate demonstrated an efficacy rate ranging from 81.7 to 90%; however, due to the lack of 100% parasite clearance the WildCat isolate is defined as having an ML-resistant phenotype. The phenotypic ML-susceptibility categorizations were upheld in the current genomic SNP analysis. 

*Dirofilaria immitis* populations are heterologous and can have a high degree of genetic variability within a population of an isolate within an individual host. ML resistance, as it is currently defined is less than 100% ML efficiency rate in in vivo chemoprophylactic studies. When in vivo clinical trials are run, only one dose rate is usually tested, i.e., that proposed for the commercial product. Therefore, it is not easy to pick up early evidence of resistance selection. To date, USA laboratory-maintained isolates are characterized as ML susceptible due to their elimination at the commercial dose rate of treatment or proposed lack of ML-drug exposure. A lack of history of prophylactics in a particular dog does not necessarily mean that the ancestors of the challenged worms had not been exposed to repeated ML chemoprophylaxis. In fact, the American Heartworm Society (AHS) recommendation that all dogs in the USA be treated 12 months of the year. As a result, *D. immitis* populations whose ancestors were truly naïve to ML prophylaxis, are becoming increasingly rare.

The current definitions used to define “ML-susceptible”, and “ML-resistance” isolates do not reflect the potential for genetically mixed populations and may not be a clear indication of an isolate’s ML-susceptibility status [27,28]. There may be *D. immitis* populations which appear susceptible at a given ML dose rate but may show a shift in susceptibility if a dose–response curve is investigated [29]. Phenotypic ML susceptibility may not correlate directly with genotypic ML susceptibility. The criteria utilized to define ML resistance in *D. immitis* may require revision to signal the genotypic variability within ML-susceptible, genetically mixed, and ML-resistant isolates and how these heterologous populations can produce variability in phenotypic assessments. 

Genomic-level testing, via the SNP molecular markers, provides key background information on the *D. immitis* isolates currently being used in laboratory and pharmaceutical research. However, little information has been documented on the potential development of ML resistance in European *D. immitis* populations. The European samples showed allele frequencies closely aligned with the *D. immitis* reference genome nDi.2.2 at the 9 SNP markers tested (Figure 2). The genotype analysis of the randomly selected European clinical samples showed that all 11 samples have genotypes consistent with ML susceptibility. The current study is a small random sampling of *D. immitis* clinical samples from a limited number of countries; however, the results of the study indicate no evidence for the development of ML resistance based on the North American SNP molecular markers in the samples tested. Following this gathering of preliminary data, a larger study across more geographical locations in canines with detailed treatment histories should be considered for conclusive evidence.

## 4. Materials and Methods

### 4.1. USA Samples

Three USA laboratory-maintained isolates were analyzed, Berkeley, Georgia II, and WildCat (Table 1). The Berkeley *D. immitis* isolate originated in Berkeley County, South Carolina, and has been maintained since April 2014. The Georgia II isolate originated in Vidalia, Georgia and has been maintained since April 2013. The WildCat isolate originated in West Liberty, Kentucky and has been maintained since August 2012. The 3 isolates were provided by TRS Lab Inc., Athens, GA, USA. 

### 4.2. European Sample Details

The 11 randomly selected European clinical samples were collected from 7 different canine hosts (Figure 1). Samples T2, T3, and T4 were collected from a single canine in Lombardy, Italy. The dog died of unrelated causes and ML treatment history is unknown. The adult female worm samples were collected at necropsy and preserved in ethanol. Samples T9, T10, and T11 were collected from a single dog in Hungary. The ML treatment history is unknown. The adult female worm samples were collected at necropsy and preserved in ethanol. Samples C1, C2, C4, and C5 originated in hunting and guard dogs that lived in rural areas Gran Canaria, in the Canary Islands, Spain (Table 1). The mf positive blood samples were collected at a large dog shelter and there was no history of ML prophylaxis in the documentation delivered with the animals. Hunting and guard dogs traditionally do not receive dirofilariosis chemoprophylactics, and several factors influence this, including low economic and socio-cultural level [30]. The dogs, from which the Gran Canaria samples were obtained, almost certainly never received prophylaxis. Finally, sample M was collected from a Spanish Greyhound from Huelva, Andalusia, Spain adopted and relocated to Savona, Italy (Table 1). Following adoption, M was placed on 24 months of monthly ivermectin (IVM) at the standard dose rate without suppression of mf and numbers at approximately 4150 mf/mL at the time of the blood draw. However, the presence of mf is not unexpected as IVM is not a registered microfilaricidal.

### 4.3. Sample Processing and DNA Extraction

The canine venous blood samples of the 3 USA laboratory-maintained isolates and the 5 European clinical blood samples were shipped to McGill University for immediate processing. The mf were extracted from the blood by filtration [8]. The blood was diluted 1:1 with NaHCO3 solution and passed through polycarbonate membrane filters (3.0 μm; 25 mm; Sterlitech^®^ Corporation, Auburn, WA, USA) to isolate mf. The 6 adult worms from Italy and Hungary canine hosts were shipped to McGill University and rehydrated in PBS prior to genomic DNA extraction.

Genomic DNA from the mf samples and the rehydrated adult worms were extracted using the QIAamp^®^ DNA Micro kit (Qiagen Inc., Toronto, ON, Canada). DNA concentrations were determined with the Quant-iT™ PicoGreen DNA Assay Kit (Invitrogen^®^, Life Technologies Inc., Burlington, ON, Canada). The 14 samples were stored at −80 °C prior to being sent to Génome Québec for sequencing.

### 4.4. SNP Markers

The 9 SNP markers used to analyze the status of ML resistance in the European samples were the top 9 markers clinically validated in 2018 to best differentiate ML-susceptible and ML-resistant phenotypes (Supplementary Material Table S1) [11]. The SNP 10 marker reported in the same study on nDi.2.2.scaf00597 at position 12,915 was not chosen for further analysis as it was not considered a reliable indicator for susceptible versus resistant genotyping.

### 4.5. Sequencing

The regions encompassing the 9 SNPs of interest were sequenced on an Illumina MiSeq Platform, at a coverage of 2000×. The Fluidigm Access Array system performed target enrichment using array-based PCR amplification of the genomic target regions. The 3 USA samples and the 11 European samples underwent parallel amplification using custom primers with added CS1 and CS2 tails, as described in Ballesteros et al. [11] (Table S1). The samples were barcoded during target enrichment which allowed for multiplexed sequencing, and adapter sequences were added during the PCR amplification reaction. The MiSeq high throughput sequencing data reported for the samples are available on the NCBI Sequence Read Archive as BAM files under the following accession numbers SAMN26854346, SAMN26854345, SAMN26854344, SAMN26854343, SAMN26854342, SAMN26854341, SAMN26854340, SAMN26854339, SAMN26854338, SAMN26854337, SAMN26854336, SAMN26854335, SAMN26854334, and SAMN26854333 in BioProject PRJNA818334.

### 4.6. Data Analysis

Trimmomatic was used to trim for minimal trailing quality (30 PHRED score) and filter for minimum read length by removing the Illumina sequencing adapters from read and adapter clippings [31]. The resulting read pairs were aligned to the *D. immitis* reference genome nDi.2.2 (http://www.nematodes.org/genomes/dirofilaria_immitis (accessed on 1 September 2019)) using BWA-mem (http://bio-bwa.sourceforge.net/ (accessed on 1 September 2019)) resulting in binary alignment map files (BAM) [32]. The alignments were processed with Picard (https://broadinstitute.github.io/picard (accessed on 1 September 2019)) for the realignment of indels, mate fixing, and marking of duplicate reads. BVATools (https://bitbucket.org/mugqic/bvatools/src (accessed on 1 September 2019)) was used to extract base frequencies at each of the 9 SNP positions and the read frequencies were assimilated to the allele frequencies (Dataset S1). Alternate alleles frequencies for the 9 SNP molecular markers were compared as half the samples were individual adult worms, for which population fixation indexes (F_ST_) could not be calculated.

## 5. Conclusions

Autochthonous transmission of canine *D. immitis* appear to be migrating from the Mediterranean and Iberian Peninsula, moving further into Central and Northern Europe, likely a result of the movement of dogs around Europe, and possibly a result of increasing mosquito populations. To date, no case of ML-resistant *D. immitis* infection has been documented in Europe. As the number of *D. immitis* infections continues to rise and spread throughout Europe, the early adoption of genotyping of clinical *D. immitis* samples could provide an early indication of the potential development of ML resistance and aid to distinguish clinical cases of heartworm infection due to ML resistance from those due to a lack of prevention or inadequate compliance, as has been seen in North America. Epidemiological surveys of *D. immitis* samples collected across Europe with diverse chemoprophylactic treatment histories from historically endemic regions of the Mediterranean, newly endemic regions such as the Balkans and Austria, and previously non-endemic regions of Northern Europe can provide insight into the genetic makeup and genetic diversity of European clinical samples.

## Figures and Tables

**Table 1:** *Dirofilaria immitis* sample identification, life stage, treatment history and origin for the US laboratory-maintained isolates and the European clinical samples which underwent MiSeq Illumina Sequencing.

Sample	Life Stage	Isolate	Dog Type	ML Treatment	Origin
**USA Laboratory-Maintained Isolates**					
WildCat	Blood mf	WildCat	Unknown	Non treated	West Liberty, KY, USA
Berkeley	Blood mf	Berkeley	Unknown	Non treated	Berkeley County, SC, USA
Georgia II	Blood mf	Georgia II	Unknown	Non treated	Vidalia, GA, USA
**European Clinical Samples**					
T2	Adult ♀	Unknown	Unknown	Unknown	Lombardy Region, Italy
T3	Adult ♀	Unknown	Unknown	Unknown	Lombardy Region, Italy
T4	Adult ♀	Unknown	Unknown	Unknown	Lombardy Region, Italy
T9	Adult ♀	Unknown	Unknown	Unknown	Hungary
T10	Adult ♀	Unknown	Unknown	Unknown	Hungary
T11	Adult ♀	Unknown	Unknown	Unknown	Hungary
C1	Blood mf	Unknown	Canary Mastiff	Non treated	Canary Island, Spain
C2	Blood mf	Unknown	Canary Mastiff	Non treated	Canary Island, Spain
C4	Blood mf	Unknown	Canary Hound	Non treated	Canary Island, Spain
C5	Blood mf	Unknown	Canary Mastiff	Non treated	Canary Island, Spain
M	Blood mf	Unknown	Spanish Greyhound	Ivermectin	^†^ Huelva, Andalusia Spain

^†^ Dog adopted to Savona, Italy.

## Data Availability

The data presented in this study are openly available on the NCBI Sequence Read Archive as BAM files under accession numbers SAMN26854346, SAMN26854345, SAMN26854344, SAMN26854343, SAMN26854342, SAMN26854341, SAMN26854340, SAMN26854339, SAMN26854338, SAMN26854337, SAMN26854336, SAMN26854335, SAMN26854334, and SAMN26854333 in BioProject PRJNA818334.

## Supplementary Materials

The following supporting information can be downloaded at: https://www.mdpi.com/article/10.3390/pathogens11080934/s1, Dataset S1: the allele count and alternative allele frequencies for the 9 SNP molecular markers when compared to the D. immitis reference genome nDi.2.2. for the three USA laboratory-maintained isolates, WildCat, Berkeley, Georgia II (Tables S1–S3) and the 11 European clinical samples, T2, T3, T4, T9, T10, T11, C1, C2, C4, C5, and M (Tables S4–S14). Data collected from genomic DNA of microfilaria or adult D. immitis samples submitted for MiSeq Illumina sequencing and calculated using BVAtools; Table S15: SNP ID, position, and primer sequences [11].
