# Supplementary material for: Dirofilaria immitis: Genotyping Randomly Selected European Clinical Samples and USA Laboratory Isolates with Molecular Markers Associated with Macrocyclic Lactone Susceptibility and Resistance
Source: Pathogens. 2022 Aug 18;11(8):934. doi: 10.3390/pathogens11080934 (PMC9415351; doi:10.3390/pathogens11080934)
Supplement: Supplementary file 1 [file pathogens-11-00934-s001.zip › CURRYetal_SNP_PathogensDataSetS1.pdf]

Dataset S1: the allele count and alternative allele frequencies for the 9 SNP molecular markers when compared to the *D. immitis* reference genome nDi.2.2.for the three US laboratory-maintained isolates, WildCat, Berkeley, Georgia II (Tables S1–S3) and the 11 European clinical samples, T2, T3, T4, T9, T10, T11, C1, C2, C4, C5, and M (Tables S4–S14). Data collected from genomic DNA of mf or adult *D. immitis* samples submitted for MiSeq Illumina sequencing and calculated using BVAtools.

Table S1. The allele count and alternative allele frequencies for the 9 SNP molecular markers when compared to the *D. immitis* reference genome nDi.2.2.for the US laboratory-maintained isolate WildCat, originating from West Liberty, Kentucky. Data collected from genomic DNA of *D. immitis* microfilaria submitted for MiSeq Illumina sequencing and calculated using BVAtools.

Scaffold	Position	Allele Counts				Allele Frequency				RefBase
WildCat		C	A	T	G	C	A	T	G	
nDi.2.2.scaf00046	76278	16914	32	15786	92	0.52	0	0.48	0	T
nDi.2.2.scaf00046	22857	19932	3	12818	2	0.61	0	0.39	0	T
nDi.2.2.scaf00046	222254	3	15258	16	17624	0	0.46	0	0.54	A
nDi.2.2.scaf00185	10639	27	8	20292	5680	0	0	0.78	0.22	T
nDi.2.2.scaf00185	62174	21126	4	6727	5	0.76	0	0.24	0	C
nDi.2.2.scaf00140	30919	15936	15029	13	14	0.51	0.58	0	0	C
nDi.2.2.scaf00005	662854	12156	63	17757	28	0.41	0	0.59	0	T
nDi.2.2.scaf00004	79766	17491	12	13709	4	0.56	0	0.44	0	T
nDi.2.2.scaf00001	466197	21	15565	10737	22	0	0.59	0.41	0	A

Table S2. The allele count and alternative allele frequencies for the 9 SNP molecular markers when compared to the *D. immitis* reference genome nDi.2.2.for the US laboratory-maintained isolate Berkeley, originating from Berkeley County, South Carolina. Data collected from genomic DNA of *D. immitis* microfilaria submitted for MiSeq Illumina sequencing and calculated using BVAtools.

Scaffold	Position	Allele Counts				Allele Frequency				RefBase
Berkeley		C	A	T	G	C	A	T	G	
nDi.2.2.scaf00046	76278	107	12	32683	147	0	0.99	0	0	T
nDi.2.2.scaf00046	22857	58	8	29550	4	0	0	1	0	T
nDi.2.2.scaf00046	222254	13	29617	22	87	0	1	0	0	A
nDi.2.2.scaf00185	10639	28	2	17661	3	0	0	1	0	T
nDi.2.2.scaf00185	62174	20241	4	1648	0	0.92	0	0.08	0	C
nDi.2.2.scaf00140	30919	22101	4415	18	17	0.83	0.17	0	0	C
nDi.2.2.scaf00005	662854	85	59	27406	54	0	0	1	0	T
nDi.2.2.scaf00004	79766	3823	6	21182	54	0.15	0	0.85	0	T
nDi.2.2.scaf00001	466197	9	24228	23	48	0	1	0	0	A

Table S3. The allele count and alternative allele frequencies for the 9 SNP molecular markers when compared to the *D. immitis* reference genome nDi.2.2.for the US laboratory-maintained isolate Georgia II, originating from Vidalia, Georgia. Data collected from genomic DNA of *D. immitis* microfilaria submitted for MiSeq Illumina sequencing and calculated using BVAtools.

Scaffold	Position	Allele Counts				Allele Frequency				RefBase
Georgia II		C	A	T	G	C	A	T	G	
nDi.2.2.scaf00046	76278	1887	12	30690	159	0.06	0	0.94	0	T
nDi.2.2.scaf00046	22857	1680	5	29397	9	0.05	0	0.95	0	T
nDi.2.2.scaf00046	222254	8	29202	25	1726	0	0.94	0	0.06	A
nDi.2.2.scaf00185	10639	40	6	20972	2	0	0	1	0	T
nDi.2.2.scaf00185	62174	20971	6	3486	4	0.86	0	0.14	0	C
nDi.2.2.scaf00140	30919	27649	23	19	3	1	0	0	0	C
nDi.2.2.scaf00005	662854	79	58	27554	41	0	0	0.99	0	T
nDi.2.2.scaf00004	79766	1615	13	26037	6	0.06	0	0.94	0	T
nDi.2.2.scaf00001	466197	5	23841	19	57	0	1	0	0	A

Table S4. The allele count and alternative allele frequencies for the 9 SNP molecular markers when compared to the *D. immitis* reference genome nDi.2.2.for European clinical sample T2, originating from the Lombardy Region, Italy. Data collected from genomic DNA of *D. immitis* adult female submitted for MiSeq Illumina sequencing and calculated using BVAtools.

Scaffold	Position	Allele Counts				Allele Frequency				RefBase
T2		C	A	T	G	C	A	T	G	
nDi.2.2.scaf00046	76278	80	31	27479	147	0	0	0.99	0.01	T
nDi.2.2.scaf00046	22857	54	16	27125	3	0	0	1	0	T
nDi.2.2.scaf00046	222254	16	26194	25	77	0	1	0	0	A
nDi.2.2.scaf00185	10639	29	13	16373	2	0	0	1	0	T
nDi.2.2.scaf00185	62174	19972	6	18	3	1	0	0	0	C
nDi.2.2.scaf00140	30919	25084	16	21	3	1	0	0	0	C
nDi.2.2.scaf00005	662854	68	72	24346	41	0	0	0.99	0	T
nDi.2.2.scaf00004	79766	85	14	22694	4	0	0	1	0	T
nDi.2.2.scaf00001	466197	13	21079	9	59	0	1	0	0	A

Table S5. The allele count and alternative allele frequencies for the 9 SNP molecular markers when compared to the *D. immitis* reference genome nDi.2.2.for European clinical sample T3, originating from the Lombardy Region, Italy. Data collected from genomic DNA of *D. immitis* adult female submitted for MiSeq Illumina sequencing and calculated using BVAtools.

Scaffold	Position	Allele Counts				Allele Frequency				RefBase
T3		C	A	T	G	C	A	T	G	
nDi.2.2.scaf00046	76278	114	15	26392	127	0	0	0.99	0	T
nDi.2.2.scaf00046	22857	73	14	27048	2	0	0	1	0	T
nDi.2.2.scaf00046	222254	10	23364	14	82	0	1	0	0	A
nDi.2.2.scaf00185	10639	18	10	11827	5	0	0	1	0	T
nDi.2.2.scaf00185	62174	18757	5	10	3	1	0	0	0	C
nDi.2.2.scaf00140	30919	21713	10	18	5	1	0	0	0	C
nDi.2.2.scaf00005	662854	95	44	25257	54	0	0	0.99	0	T
nDi.2.2.scaf00004	79766	75	4	18803	4	0	0	1	0	T
nDi.2.2.scaf00001	466197	2	22252	21	55	0	1	0	0	A

Table S6. The allele count and alternative allele frequencies for the 9 SNP molecular markers when compared to the *D. immitis* reference genome nDi.2.2.for European clinical sample T4, originating from the Lombardy Region, Italy. Data collected from genomic DNA of *D. immitis* adult female submitted for MiSeq Illumina sequencing and calculated using BVAtools.

Scaffold	Position	Allele Counts				Allele Frequency				RefBase
T4		C	A	T	G	C	A	T	G	
nDi.2.2.scaf00046	76278	80	14	29237	125	0	0	0.99	0	T
nDi.2.2.scaf00046	22857	79	3	31371	8	0	0	1	0	T
nDi.2.2.scaf00046	222254	3	28493	30	88	0	1	0	0	A
nDi.2.2.scaf00185	10639	27	4	13618	7	0	0	1	0	T
nDi.2.2.scaf00185	62174	21689	7	53	0	1	0	0	0	C
nDi.2.2.scaf00140	30919	25533	30	14	2	1	0	0	0	C
nDi.2.2.scaf00005	662854	88	70	27343	40	0	0	0.99	0	T
nDi.2.2.scaf00004	79766	74	6	22054	2	0	0	1	0	T
nDi.2.2.scaf00001	466197	13	23673	32	47	0	1	0	0	A

Table S7. The allele count and alternative allele frequencies for the 9 SNP molecular markers when compared to the *D. immitis* reference genome nDi.2.2.for European clinical sample T9, originating from Hungary. Data collected from genomic DNA of *D. immitis* adult female submitted for MiSeq Illumina sequencing and calculated using BVAtools.

Scaffold	Position	Allele Counts				Allele Frequency				RefBase
T9		C	A	T	G	C	A	T	G	
nDi.2.2.scaf00046	76278	100	11	26435	127	0	0	0.99	0	T
nDi.2.2.scaf00046	22857	75	12	31584	3	0	0	1	0	T
nDi.2.2.scaf00046	222254	16	26722	17	102	0	0.99	0	0	A
nDi.2.2.scaf00185	10639	18	0	10045	1	0	0	1	0	T
nDi.2.2.scaf00185	62174	20368	7	11	0	1	0	0	0	C
nDi.2.2.scaf00140	30919	22804	14	8	4	1	0	0	0	C
nDi.2.2.scaf00005	662854	87	63	27962	51	0	0	0.99	0	T
nDi.2.2.scaf00004	79766	128	8	20933	5	0.01	0	0.99	0	T
nDi.2.2.scaf00001	466197	7	24316	18	31	0	1	0	0	A

Table S8. The allele count and alternative allele frequencies for the 9 SNP molecular markers when compared to the *D. immitis* reference genome nDi.2.2.for European clinical sample T10, originating from Hungary. Data collected from genomic DNA of *D. immitis* adult female submitted for MiSeq Illumina sequencing and calculated using BVAtools.

Scaffold	Position	Allele Counts				Allele Frequency				RefBase
T10		C	A	T	G	C	A	T	G	
nDi.2.2.scaf00046	76278	67	12	22664	123	0	0	0.99	0.01	T
nDi.2.2.scaf00046	22857	90	7	27415	2	0	0	1	0	T
nDi.2.2.scaf00046	222254	4	22890	27	110	0	0.99	0	0	A
nDi.2.2.scaf00185	10639	18	5	10640	2	0	0	1	0	T
nDi.2.2.scaf00185	62174	18442	2	23	2	1	0	0	0	C
nDi.2.2.scaf00140	30919	21654	23	13	2	1	0	0	0	C
nDi.2.2.scaf00005	662854	86	53	22887	45	0	0	0.99	0	T
nDi.2.2.scaf00004	79766	50	15	17788	6	0	0	1	0	T
nDi.2.2.scaf00001	466197	4	19642	9	46	0	1	0	0	A

Table S9. The allele count and alternative allele frequencies for the 9 SNP molecular markers when compared to the *D. immitis* reference genome nDi.2.2.for European clinical sample T11, originating from Hungary. Data collected from genomic DNA of *D. immitis* adult female submitted for MiSeq Illumina sequencing and calculated using BVAtools.

Scaffold	Position	Allele Counts				Allele Frequency				RefBase
T11		C	A	T	G	C	A	T	G	
nDi.2.2.scaf00046	76278	56	3	22645	111	0	0	0.99	0	T
nDi.2.2.scaf00046	22857	30	1	25528	4	0	0	1	0	T
nDi.2.2.scaf00046	22857	30	1	25528	4	0	0	1	0	T
nDi.2.2.scaf00185	10639	27	1	13540	1	0	0	1	0	T
nDi.2.2.scaf00185	62174	18542	3	15	1	1	0	0	0	C
nDi.2.2.scaf00140	30919	18340	11	15	1	1	0	0	0	C
nDi.2.2.scaf00005	662854	50	51	23208	35	0	0	0.99	0	T
nDi.2.2.scaf00004	79766	45	2	18282	1	0	0	1	0	T
nDi.2.2.scaf00001	466197	6	19183	11	31	0	1	0	0	A

Table S10. The allele count and alternative allele frequencies for the 9 SNP molecular markers when compared to the *D. immitis* reference genome nDi.2.2.for European clinical sample C1, originating from the Canary Islands, Spain. Data collected from genomic DNA of *D. immitis* microfilaria submitted for MiSeq Illumina sequencing and calculated using BVAtools.

Scaffold	Position	Allele Counts				Allele Frequency				RefBase
C1		C	A	T	G	C	A	T	G	
nDi.2.2.scaf00046	76278	80	9	20269	94	0	0	0.99	0	T
nDi.2.2.scaf00046	22857	63	15	21227	4	0	0	1	0	T
nDi.2.2.scaf00046	222254	6	19732	32	102	0	0.99	0	0	A
nDi.2.2.scaf00185	10639	12	7	9602	3	0	0	1	0	T
nDi.2.2.scaf00185	62174	15604	5	10	0	1	0	0	0	C
nDi.2.2.scaf00140	30919	19144	13	9	4	1	0	0	0	C
nDi.2.2.scaf00005	662854	47	43	20596	35	0	0	0.99	0	T
nDi.2.2.scaf00004	79766	59	8	16062	1	0	0	1	0	T
nDi.2.2.scaf00001	466197	3	17385	11	50	0	1	0	0	A

Table S11. The allele count and alternative allele frequencies for the 9 SNP molecular markers when compared to the *D. immitis* reference genome nDi.2.2.for European clinical sample C2, originating from the Canary Islands, Spain. Data collected from genomic DNA of *D. immitis* microfilaria submitted for MiSeq Illumina sequencing and calculated using BVAtools.

Scaffold	Position	Allele Counts				Allele Frequency				RefBase
C2		C	A	T	G	C	A	T	G	
nDi.2.2.scaf00046	76278	75	17	25099	122	0	0	0.99	0	T
nDi.2.2.scaf00046	22857	71	10	25172	0	0	0	1	0	T
nDi.2.2.scaf00046	222254	7	23996	29	983	0	0.96	0	0.04	A
nDi.2.2.scaf00185	10639	28	10	14837	9	0	0	1	0	T
nDi.2.2.scaf00185	62174	193170	22	0	0	1	0	0	0	C
nDi.2.2.scaf00140	30919	22151	28	14	4	1	0	0	0	C
nDi.2.2.scaf00005	662854	64	71	22511	39	0	0	0.99	0	T
nDi.2.2.scaf00004	79766	904	1	18802	5	0.05	0	0.95	0	T
nDi.2.2.scaf00001	466197	2	19345	12	54	0	1	0	0	A

Table S12. The allele count and alternative allele frequencies for the 9 SNP molecular markers when compared to the *D. immitis* reference genome nDi.2.2.for European clinical sample C4, originating from the Canary Islands, Spain. Data collected from genomic DNA of *D. immitis* microfilaria submitted for MiSeq Illumina sequencing and calculated using BVAtools.

Scaffold	Position	Allele Counts				Allele Frequency				RefBase
C4		C	A	T	G	C	A	T	G	
nDi.2.2.scaf00046	76278	36	8	7959	47	0	0	0.99	0.01	T
nDi.2.2.scaf00046	22857	29	0	12454	5	0	0	1	0	T
nDi.2.2.scaf00046	222254	0	8640	9	37	0	0.99	0	0	A
nDi.2.2.scaf00185	10639	8	2	2232	2	0	0	0.99	0	T
nDi.2.2.scaf00185	62174	6922	4	16	0	1	0	0	0	C
nDi.2.2.scaf00140	30919	9696	14	6	2	1	0	0	0	C
nDi.2.2.scaf00005	662854	59	25	12980	33	0	0	0.99	0	T
nDi.2.2.scaf00004	79766	21	5	4362	1	0	0	0.99	0	T
nDi.2.2.scaf00001	466197	3	9370	5	23	0	1	0	0	A

Table S13. The allele count and alternative allele frequencies for the 9 SNP molecular markers when compared to the *D. immitis* reference genome nDi.2.2.for European clinical sample C5, originating from the Canary Islands, Spain. Data collected from genomic DNA of *D. immitis* microfilaria submitted for MiSeq Illumina sequencing and calculated using BVAtools.

Scaffold	Position	Allele Counts				Allele Frequency				RefBase
C5		C	A	T	G	C	A	T	G	
nDi.2.2.scaf00046	76278	49	10	19675	80	0	0	0.99	0	T
nDi.2.2.scaf00046	22857	27	4	19946	3	0	0	1	0	T
nDi.2.2.scaf00046	222254	4	18179	17	1576	0	0.92	0	0.08	A
nDi.2.2.scaf00185	10639	45	7	14279	10	0	0	1	0	T
nDi.2.2.scaf00185	62174	15735	0	16	1	1	0	0	0	C
nDi.2.2.scaf00140	30919	18567	15	19	3	1	0	0	0	C
nDi.2.2.scaf00005	662854	51	47	18298	25	0	0	0.99	0	T
nDi.2.2.scaf00004	79766	1351	2	16119	5	0.08	0	0.92	0	T
nDi.2.2.scaf00001	466197	3	15783	13	31	0	1	0	0	A

Table S14. The allele count and alternative allele frequencies for the 9 SNP molecular markers when compared to the *D. immitis* reference genome nDi.2.2.for European clinical sample M, from Huelva, Andulusia, Spain , adopted and relocated to Savona, Italy. Data collected from genomic DNA of *D. immitis* microfilaria submitted for MiSeq Illumina sequencing and calculated using BVAtools.

Scaffold	Position	Allele Counts				Allele Frequency				RefBase
M		C	A	T	G	C	A	T	G	
nDi.2.2.scaf00046	76278	116	17	27101	119	0	0	0.99	0	T
nDi.2.2.scaf00046	22857	61	8	28136	10	0	0	1	0	T
nDi.2.2.scaf00046	222254	8	25508	40	105	0	0.99	0	0	A
nDi.2.2.scaf00185	10639	28	5	13581	5	0	0	1	0	T
nDi.2.2.scaf00185	62174	20278	4	18	1	1	0	0	0	C
nDi.2.2.scaf00140	30919	23777	15	27	0	1	0	0	0	C
nDi.2.2.scaf00005	662854	72	45	26547	65	0	0	0.99	0	T
nDi.2.2.scaf00004	79766	118	5	20352	4	0.01	0	0.99	0	T
nDi.2.2.scaf00001	466197	5	22744	18	79	0	1	0	0	A
