# Supplementary material for: Dirofilaria immitis: Genotyping Randomly Selected European Clinical Samples and USA Laboratory Isolates with Molecular Markers Associated with Macrocyclic Lactone Susceptibility and Resistance
Source: Pathogens. 2022 Aug 18;11(8):934. doi: 10.3390/pathogens11080934 (PMC9415351; doi:10.3390/pathogens11080934)
Supplement: Supplementary file 1 [file pathogens-11-00934-s001.zip › CURRYetal_SNP_PathogensTableS15.pdf]

Table S15 SNP ID position and primer sequences [11]

ID	Scaffold	Position	Primer Name & Sequence
SNP 1	nDi.2.2.scaf00001	466197	scaf00001-466197-CS1F ACACTGACGACATGGTTCTACATTCTATCGAAAACCTTCCAG<br>scaf00001-466197-CS2R TACGGTAGCAGAGACTTGGTCTAGGTTGCAAAAGTTGCAATG
SNP 2	nDi.2.2.scaf00004	79766	scaf00004-79766-CS1F ACACTGACGACATGGTTCTACACGTGACTAAAAGAATAGTG<br>scaf00004-79766-CS2R TACGGTAGCAGAGACTTGGTCTCAATTTAGGGATATGACACAG
SNP 3	nDi.2.2.scaf00005	662854	scaf00005-662854-CS1F ACACTGACGACATGGTTCTACAGTTATTTGCACTACTCTCCC<br>scaf00005-662854-CS2R TACGGTAGCAGAGACTTGGTCTTGGCGTACTGATCACATTGG
SNP 4	nDi.2.2.scaf00046	22857	scaf00046-22857-CS1F ACACTGACGACATGGTTCTACACGAGGTAAAGCACACAGAAG<br>scaf00046-22857-CS2R TACGGTAGCAGAGACTTGGTCTCAACAAAATGCCGCAGATGG
SNP 5	nDi.2.2.scaf00046	76278	scaf00046-76278-CS1F ACACTGACGACATGGTTCTACAGGCCAATAAATAAAGGCTA<br>scaf00046-76278-CS2R TACGGTAGCAGAGACTTGGTCTGTTTTCTGGAATTATCAGAC
SNP 6	nDi.2.2.scaf00046	222254	scaf00046-222254-CS1F ACACTGACGACATGGTTCTACACATCGTTGTCAACTTCCTGC<br>scaf00046-222254-CS2R TACGGTAGCAGAGACTTGGTCTGAAATTTGAAAATGGGTACT
SNP 7	nDi.2.2.scaf00140	30919	scaf00140-30919-CS1F ACACTGACGACATGGTTCTACACGAAGAAGAAACTTTTCGGG<br>scaf00140-30919-CS2R TACGGTAGCAGAGACTTGGTCTGTACAATTAATTGCTGTTCGC
SNP 8	nDi.2.2.scaf00185	10639	scaf00185-10639-CS1F ACACTGACGACATGGTTCTACAACGCAGGAAAGCTTTAATGG<br>scaf00185-10639-CS2R TACGGTAGCAGAGACTTGGTCTATCATCATTTTATCAATTCC
SNP 9	nDi.2.2.scaf00185	62174	scaf00185-62174-CS1F ACACTGACGACATGGTTCTACATCGATCATTTAGTAACAACG<br>scaf00185-62174-CS2R TACGGTAGCAGAGACTTGGTCTTTGCGTTACAGCGCCAAATC
